# Health of nursing professionals: workload during the COVID-19 pandemic

**DOI:** 10.47626/1679-4435-2020-600

**Published:** 2021-03-03

**Authors:** Diego de Oliveira Souza

**Affiliations:** Complexo de Ciências Médicas e Enfermagem, Universidade Federal de Alagoas, Arapiraca, AL, Brazil

**Keywords:** coronavirus, nursing, pandemic, occupational health, public health

## Abstract

The aim of this study was to examine the workload of nurses and its dynamics during the first months (January to April 2020) of the coronavirus disease 2019 (COVID-19) pandemic. An integrative review of the National Library of Medicine (PubMed) and Virtual Health Library was conducted using the keywords *coronavirus and nursing*. Results were analyzed based on the theories of Laurell & Noriega. The 9 articles reviewed emphasized the role of the biological workload associated with COVID-19, in addition to that of sources of psychological workload such as the fear of contamination, the burden of responsibility and concerns about parents. The successful management of the pandemic depends on our ability to mitigate the effects of this workload, especially in light of the quantitative and qualitative importance of nursing in health care institutions.

## INTRODUCTION

The year 2020 was marked around the world by the novel coronavirus disease (COVID-19). The outbreak, which began in late December 2019 in Wuhan, China, was declared a pandemic by the World Health Organization (WHO) on March 11. The number of confirmed cases and deaths increased rapidly, and by April 30 2020, a total of 3,090,445 cases and 217,769 deaths had been reported worldwide.^1^ The pandemic represented a critical challenge for frontline workers, especially those in the health care sector. On April 1 2020, Spain reported 6,500 cases of COVID-19 among health care workers, while Italy reported 6,200, and China, 3,300; however, these figures are likely to be underestimated due to underreporting and testing delays. Health care workers are thought to account for 4 to 12% of confirmed cases of COVID-19.^2^

Nursing professionals were among the most severely affected, as they represent the largest share of the global health care workforce.^3^ In Brazil, according to the National Counsel of Nursing,^4^ the number of suspected cases in this population rose from 230 on April 5 to 4,089 (552 confirmed and over 3,500 under investigation) in only 10 days. By that date, COVID-19 had claimed the lives of 30 nursing professionals, and over the following month, this figure would increase by a factor of three, causing a total of 98 deaths in this population. The data reveal a concerning and dynamic phenomenon, demanding equally swift and comprehensive responses from science in the form of initiatives to protect health care workers, especially those in the field of nursing, who are exposed to occupational and health workloads that stem from but extend far beyond the issue of COVID-19.^3^

The concept of “workload” as described by Laurell & Noriega^5^ may shed light on the dynamics of the situation faced by nursing professionals in the first months of the pandemic. It is important to note that this theory does not see occupational risk as a static concept, or an inherent part of the work process, as it is often described.^5^ Instead, workload is viewed as a set of dynamic components of the work process and environment, arising from different sources, which can be internal or external to the worker.^5^ External sources of strain include physical variables, such as radiation and variations in temperature or atmospheric pressure; chemical variables such as the handling of acids, solvents or drugs; biological variables such as viruses and bacteria; and mechanical variables, present in situations involving accident risks.^5^

Internal sources of strain, on the other hand, include physiological variables, often related to ergonomic factors such as poor posture, overexertion or excess weight bearing; and psychological variables which influence psycho-emotional well-being, such as excessive working hours, constant demands for attention or problems in interpersonal relationships.^5^ In light of these observations, this review aimed to examine the workload of nursing activities during the COVID-19 pandemic, focusing in the first quarter of 2020.

## METHODS

An integrative literature review was performed according to the following steps: development of research question, literature search/article selection, data collection, critical assessment of results, discussion and presentation of the integrative review. The process was guided by the following research question: what are the sources of burden in nursing activities and how did they evolve in the first months of the COVID-19 pandemic? This was addressed by searching the National Library of Medicine (PubMed) and Virtual Health Library (VHL) using the keywords *coronavirus and nursing*, combined with the Boolean operator and. An additional search was performed with the term *occupational health* added to the aforementioned keywords using the and operator; however, this yielded no results, and the additional term was removed from the search strategy.

Eligible articles were published between January and April 2020 in English, Portuguese and Spanish. Titles and abstracts were screened to select the articles that answered the research question. Duplicates, editorials, letters and commentaries were excluded. The articles selected were then retrieved in full text, and read to extract information on the following variables: authors, journal, objective, type of study and results.

The first stage in the analysis involved classifying articles according to their levels of evidence (LOE), which the Agency for Healthcare and Research and Quality (AHRQ) describes as follows: 1) systematic review or meta-analysis, 2) randomized clinical trial, 3) non-randomized clinical trial, 4) case-control or cohort study, 5) systematic review of descriptive and qualitative studies, and 6) a single descriptive or qualitative study.^6^ This system was adapted to include a seventh level for research methods such as case studies, reports and action research, given the number and importance of these studies in exceptional situations such as the pandemic. Lastly, the results of the articles were analyzed based on the concept of workload described by Laurell & Noriega,^5^ as mentioned in the introduction of this review, before being presented and critically discussed.

## RESULTS

The keywords *coronavirus and nursing* retrieved 209 articles in PubMed and 159 in the VHL. The results were then filtered to include only those published in 2020 (PubMed = 86 and HVL = 54), then screened to select only those that addressed the research question (PubMed = 7 and VHL = 7). The exclusion of editorials, letters and commentaries left 6 of the articles found in PubMed and 4 in VHL. One article was duplicated across databases. The final sample therefore consisted of 9 articles, one of which was present in both databases, while 5 were found only in PubMed and 3 in the VHL. The characteristics of the articles included in the review are shown in Chart 1.

**Chart 1:** Characteristics of included studies (January-April 2020)

Authors	Journal	Objective	Type of study	LOE
Kang et al.^7^	*Brain, Behavior, and Immunity*	To assess the mental health status and psychological care needs of medical and nursing professionals in Wuhan, as well as the efficacy of available psychological interventions.	Cross-sectional quantitative	6
Liu et al.^8^	*International Journal of Nursing Sciences*	To describe the implementation of an emergency management system for human resources and supplies in the nursing department of a large general hospital during the COVID-19 pandemic.	Report	7
Lai et al.^9^	*JAMA Network Open*	To assess mental health outcomes and associated factors in health care workers treating patients exposed to COVID-19 in China.	Cross-sectional quantitative	6
Rowan & Laffey^10^	*Science of The Total Environment*	To analyze the strategies used in the Republic of Ireland to address the shortage of PPE during the COVID-19 pandemic.	Case study	7
Wang et al.^11^	*International Journal of Nursing Sciences*	To summarize a series of contingency management strategies implemented by a nursing department to facilitate the centralized treatment of patients with COVID-19.	Report	7
Wu et al.^12^	*Journal of Pain and Symptom Management*	To compare the prevalence of burnout in frontline physicians and nurses to that of individuals working in regular hospital departments.	Cross-sectional quantitative	6
Burch^13^	*British Journal of Nursing*	To communicate the risks and consequences of COVID-19 to gastroenterology nurses.	Report	7
Qian et al.^14^	*International Journal of Nursing Sciences*	To evaluate the implementation of safety management strategies for the collection of nasopharyngeal swabs from patients with suspected COVID-19 in a reference hospital.	Action research	7
Mo et al.^15^	*Journal of Nursing Management*	To investigate occupational stress and associated factors in Chinese nurses working in the front lines of the COVID-19 pandemic.	Cross-sectional quantitative	6

COVID-19 = coronavirus disease 2019; PPE = personal protective equipment; LOE = level of evidence; SARS-CoV-2 = severe acute respiratory syndrome coronavirus 2.

As can be seen in the chart, only one journal contained more than one of the articles reviewed: the International Journal of Nursing Sciences, with three publications included in the present study. Most studies had a cross-sectional quantitative design (n = 4), which constituted the highest level of evidence identified in this review. The remaining five articles were classified in level 7, suggesting there is still limited evidence on the impact of COVID-19 on the health of nursing professionals during the first months of the pandemic. Nevertheless, the studies included in this review identified important issues regarding work burden which may be analyzed in greater depth by future studies. The results emphasized three main sources of work burden: the severe acute respiratory syndrome coronavirus 2 (SARS-CoV-2) itself, which is a source of biological workload; the dynamics of the pandemic as a source of psychological workload; and organizational issues that, if left unresolved, can cause many different types of stress.

Chart 2 contains information on articles that focused more closely on biological stresses experienced by nursing professionals during the COVID-19 pandemic.

**Chart 2:** Results of included studies on biological workload (January-April 2020)

Authors	Results
Burch^13^	In addition to the risk of airway transmission, gastroenterology nurses are vulnerable to contamination during endoscopy or intestinal procedures, since the virus has been reported to remain viable in feces for up to 33 days. The study recommended the postponement of elective procedures and an increase in the availability of (online) educational resources. Procedures that cannot be delayed should be performed using PPE and following stringent hygiene and disinfection guidelines.
Qian et al.^14^	The key points for reducing the risk of infection for the eight nurses who collected nasopharyngeal samples were: establishing a dedicated sampling room; strict sterilization of equipment and environment; training professional nurses; enhancing personal protections; standardizing methods and procedures for swab collection; and safe and timely sample submission. After collecting the nasopharyngeal samples, all nurses tested negative for SARS-CoV-2.

COVID-19 = coronavirus disease 2019; PPE = personal protective equipment; SARS-CoV-2 = severe acute respiratory syndrome coronavirus 2.

In addition to representing a source of biological workload, the changing nature of the pandemic is a source of psychological stress, especially for those in constant contact with infected individuals. The psychoemotional burden experienced by nursing professionals was addressed in the articles described in Chart 3.

**Chart 3:** Results of included studies on psychological workload (January-April 2020)

Authors	Results
Kang et al.^7^	Of 994 health care workers (183 physicians and 811 nurses) in Wuhan, 36.9% had mental disorders, 34.4% reported mild distress, 22.4% reported moderate distress, and 6.2% reported severe distress at the start of the pandemic. Young women were overrepresented among nurses, and bore a significant share of this burden. The study found that 36.3% of participants had accessed psychological support materials, 50.4% had accessed psychological support via social media and 17.5% had participated in psychotherapy.
Lai et al.^9^	The study involved a sample of 1,257 workers, including 764 (60.8%) nurses and 493 (39.2%) physicians; 760 (60.5%) worked in hospitals in Wuhan; and 522 (41.5%) were frontline workers. A considerable number of participants had symptoms of depression (50.4%), anxiety (44.6%), insomnia (34%) and distress (71.5%). The most severe symptoms were seen in nurses, women, frontline workers and those working in Wuhan, China. Workers outside of Hubei province had a lower risk of distress than those working in Wuhan. Frontline workers involved in the diagnosis, treatment and care of patients with COVID-19 were at higher risk of developing depression symptoms.
Wu et al.^12^	Physicians and nurses (n = 220) in departments that did not see patients with COVID-19 were also at risk of burnout. In fact, frontline workers treating patients with COVID-19 had lower levels of burnout than those in regular hospital wards (13 vs 39%, p < 0.0001).
Mo et al.^15^	In a sample of 180 nurses, significant correlations were seen between scores on the Stress Overload Scale (39.91±12.92) and the Self-assessed Anxiety Scale (32.19±7.56), (r = 0.676, p < 0.05). Multivariate regression showed that weekly work hours, anxiety and worries about their parents were the main contributors to stress among nurses (p = 0.000, 0.048 and 0.000, respectively).

COVID-19 = coronavirus disease 2019.

Organizational responses involving management, engineering, protocols and procedures play a crucial role in mitigating the effects of various types of workload. Mechanical and physiological burden almost always comes from disordered processes or systems. The impact of biological, chemical and physical burdens can also be amplified by a lack of organizational rigor. Chart 4 presents the results of studies that focused on this particular issue.

**Chart 4:** Results of included studies on organizational issues

Authors	Results
Liu et al.^8^	The implementation of an emergency management plan for human resources and supplies had a positive effect on the response capacity of the nursing team. The plan included measures such as the allocation of additional nurses to relieve overburdened workers, the dynamic deployment of human resources, pre-service training and assessment, focused supervision, positive encouragement and a technical-scientific approach to supply management.
Rowan & Laffey^10^	The solutions implemented in the Republic of Ireland included the use of smart communication channels to improve the supply chain of PPE, bespoke production and, in exceptional cases, the use of sterilization or high-level disinfection for reprocessing. The reprocessing of PPE should consider the composition of the materials, post-treatment functionality and appropriate disinfection. The authors also emphasize the importance of following manufacturers' instructions and sustainability guidelines.
Wang et al.^11^	The contingency management strategies adopted by the nursing department included early warnings for prevention and control; vertical command and horizontal coordination; dynamic allocation of human resources, with a special focus on in-hospital fever clinics, isolation wards, ICUs, referral sectors and the admission of critical patients. Five special teams were also formed and put in charge of: 1) training and examination; 2) management and supervision; 3) psychological support; 4) logistical support; and 5) reporting and publicity. The implementation of these strategies allowed for the safe, efficient, timely, organized and sustainable provision of centralized treatment.

ICU = intensive care unit; PPE = personal protective equipment.

## DISCUSSION

The investigation of work burden in individuals facing an epidemic or pandemic highlighted the role of biological workload. The concern about these issues was reflected in the articles reviewed by the testing of nursing professionals for SARS-CoV-2,^14^ and the identification of high-risk activities (collection of nasopharyngeal specimens)^14^ and means of transmission (in gastroenterological procedures).^15^

The impact of biological workload on nursing professionals therefore takes a central role in the process investigated in the present review; nevertheless, these issues must be investigated within an historical and social context, as noted by Laurell & Noriega,^5^ who emphasize the dynamic and socially determined nature of work burden. As such, the research question cannot be reduced to its biological dimension; instead, the clearly evident biological factors must be viewed as a distinct manifestation of the social determination of health.^16^ This perspective requires the consideration, among other things, of the historical issues that lead workers to assume particular roles, the economic and political determinants of better or worse preparedness to face COVID-19, and even the history of work relationships within each institution.

This theoretical perspective also draws attention to psychological burdens, with incidents faced by frontline workers^7,9,12,15^ seen as equally or more important than biological factors, and extending their reach to those who are not in direct contact with COVID-19 patients.^12^ The fear of contamination was a significant source of psychological workload, which clashed with the responsibility to contribute to the fight against the greatest public health challenge in decades, explaining the frequency of mental disorders,^7^ anxiety, distress, insomnia and depression among nursing professionals in Wuhan.^9^ Other sources of workload mentioned in the studies reviewed included concerns about their parents and exhaustion due to long working hours,^15^ as well as burnout, which is directly and indirectly associated with working in health care institutions.^12^

The organizational factors discussed in the articles included emergency resource management, the organization and supervision of work processes,^8,11^ and the creation of specialized teams,^11^ all of which can decrease the chance of contamination and the effects of biological workloads such as exertion and overwork, in addition to increasing psychological comfort. Personal protective equipment (PPE) has also emerged as a major issue during the pandemic; its absence^10^ may be a source of mechanical stress due to the increased risk of accidents and subsequent contamination, which in turn magnifies the effects of biological workload, and generates insecurity, a form of psychological stress. The frequent cleaning, disinfection and sterilization, sometimes in the context of PPE reprocessing,^10^ can also be viewed as a source of chemical or physical workload depending on the activities involved.^17^

**Figure 1:**
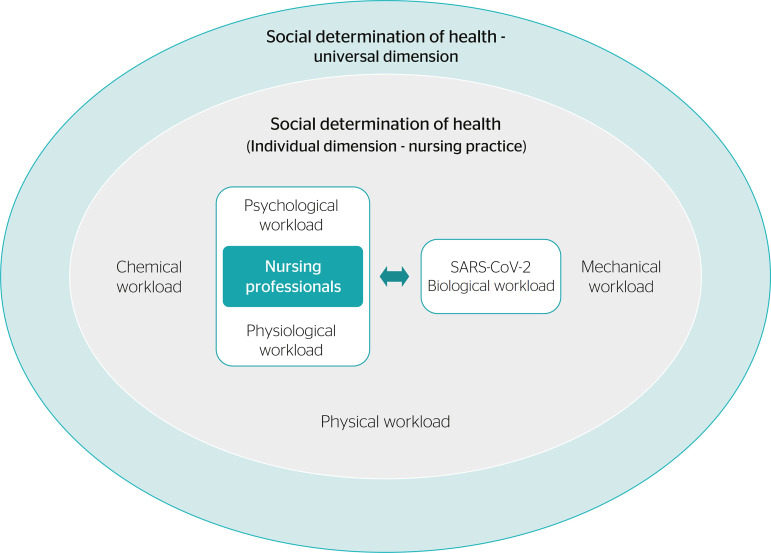
Dynamics of work burden among nursing professionals during the coronavirus disease 2019 (COVID-19) pandemic. SARS-CoV-2 = severe acute respiratory syndrome coronavirus 2.

The dynamics of nursing practice are a part of a larger process, which involves global social issues such as the movement of people and objects, political decisions on health and the economy, rapid scientific responses, and the consistency of epidemiological information. These large-scale issues must be considered in order to understand the process of social determination of health, which has particular as well as universal dimensions.^16^ This process is illustrated in Figure 1.

These observations show how one salient factor (i.e., exposure to the novel coronavirus) can trigger or reinforce several other types of workload. These dynamic^^5^^ workloads interact with one another, and with elements both intrinsic and extrinsic to the process of work, contributing to the burnout caused by COVID-19 itself and its psycho-emotional implications. Part of this burden is at least partly present in the everyday practice of nursing (and, indeed, of health care as a whole), even in the absence of a pandemic, but is amplified by the tensions of this exceptional situation, especially due to the particularities of nursing work, which demands close contact with patients.

Previous studies have analyzed the work burden of nursing professionals, especially in hospital settings. Carvalho et al.^18^ noted that the most common issues affecting these workers are viruses as a source of biological workload, sustained tension as a form of psychological workload, and physical exertion as a source of physiological workload. Psychological workload was examined in greater detail by Secco et al.,^19^ who highlighted the impact of exposure to pain and death, which apply to the context of COVID-19, where these effects are magnified by the fear of contamination. Mininel et al.,^20^ in turn, identified sources of psychological workload such as the fast pace of work, the lack of collective defense and the constant demands for attention, all of which are amplified in a context like that of the pandemic.

The understanding of the dynamics of work burden and its peculiarities in the context of the pandemic is crucial for the formulation and execution of actions to preserve the health of nursing professionals facing this global challenge. Future analysis of the impact of the pandemic will allow for a reassessment of these reflections and further additions to the model described.

## CONCLUSION

The first months of the COVID-19 pandemic shed light on the work burden of nursing professionals, with psychological workload playing an important role, even though a source of biological workload - the coronavirus itself - is at the core of this dynamic framework. The successful management of the pandemic depends on the ability to mitigate these workloads, eliminating them at their source in the process of work, but also acting on the universal dynamics of social determination, considering the role of nursing within health care institutions.

The review identified important strategies to cope with burden, especially that arising from the biological workloads associated with the risk of infection. The amplification of these measures requires well-prepared health systems, adequate working conditions, consistent *occupational health* surveillance programs and the strengthening of science to provide swift and focused responses.
